# Risk Factors for Transient and Permanent Hypoparathyroidism Following Thyroidectomy: A Comprehensive Review

**DOI:** 10.7759/cureus.66551

**Published:** 2024-08-09

**Authors:** Maulik Maheshwari, Imran Ali Khan

**Affiliations:** 1 General Surgery, Jawaharlal Nehru Medical College, Datta Meghe Institute of Higher Education and Research, Wardha, IND

**Keywords:** surgical complications, permanent hypocalcemia, transient hypocalcemia, parathyroid glands, hypoparathyroidism, thyroidectomy

## Abstract

Hypoparathyroidism is a common complication following thyroidectomy, resulting in significant disturbances in calcium homeostasis due to low parathyroid hormone (PTH) levels. This comprehensive review examines the risk factors associated with transient and permanent hypoparathyroidism post-thyroidectomy, emphasizing surgical, patient-related, and perioperative factors. Transient hypoparathyroidism, characterized by temporary hypocalcemia resolving within weeks to months, is often managed with short-term calcium and vitamin D supplementation. In contrast, permanent hypoparathyroidism persists beyond six months post-surgery, necessitating lifelong supplementation and potentially PTH replacement therapy. The review delves into the anatomy and physiology of the parathyroid glands, mechanisms leading to hypoparathyroidism, and incidence rates. Surgical factors such as the extent of thyroidectomy, surgeon expertise, and intraoperative parathyroid gland preservation are critical in determining the risk of hypoparathyroidism. Patient factors, including age, sex, pre-existing conditions, and perioperative management, influence outcomes. Diagnostic and monitoring strategies, along with management protocols for both transient and permanent hypoparathyroidism, are discussed. Prevention strategies, emerging research, future surgical techniques, and intraoperative monitoring directions are highlighted to improve clinical outcomes. This review aims to enhance understanding, inform surgical practices, and optimize postoperative care to minimize the incidence and impact of hypoparathyroidism in thyroidectomy patients.

## Introduction and background

Thyroidectomy, the surgical removal of all or part of the thyroid gland, is a common procedure performed for various indications, including thyroid cancer, benign thyroid nodules, goiter, and hyperthyroidism [1]. The procedure may involve a total thyroidectomy, which is the complete removal of the thyroid gland, or a subtotal/partial thyroidectomy, which involves removing a portion of the gland. Despite advancements in surgical techniques and perioperative care, thyroidectomy carries the risk of complications, with hypoparathyroidism being particularly significant due to its impact on calcium homeostasis [2]. The parathyroid glands, small endocrine glands typically numbering four and located posterior to the thyroid gland, play a crucial role in regulating calcium levels in the blood through parathyroid hormone (PTH) secretion. PTH increases blood calcium levels by stimulating calcium release from bones, enhancing calcium absorption in the intestines, and promoting calcium reabsorption in the kidneys. Given their essential function, the inadvertent damage or removal of these glands during thyroidectomy can lead to significant disturbances in calcium metabolism [3].

Hypoparathyroidism following thyroidectomy is characterized by low levels of PTH, leading to hypocalcemia. This condition is categorized into transient and permanent forms based on the duration and persistence of hypocalcemia. Transient hypoparathyroidism occurs when hypocalcemia is temporary, typically resolving within a few weeks to months post-surgery as the parathyroid glands recover their function [4]. It is usually managed with short-term calcium and vitamin D supplementation. In contrast, permanent hypoparathyroidism is diagnosed when hypocalcemia persists beyond six months postoperatively, indicating that the parathyroid glands have not regained function. Patients with permanent hypoparathyroidism require lifelong calcium and vitamin D supplementation and, in some cases, PTH replacement therapy [5]. This comprehensive review aims to systematically examine the risk factors associated with the development of transient and permanent hypoparathyroidism following thyroidectomy. By analyzing surgical, patient-related, and perioperative factors, this review provides a detailed understanding of the mechanisms leading to hypoparathyroidism, identifies strategies for prevention and management, and highlights areas for future research. Ultimately, this review aims to enhance clinical outcomes by informing surgical practices and postoperative care protocols to minimize the incidence and impact of hypoparathyroidism in patients undergoing thyroidectomy.

## Review

Anatomy and physiology of the parathyroid glands

The parathyroid glands are small but essential endocrine glands that play a crucial role in regulating calcium homeostasis in the body. Most individuals have four parathyroid glands located on the posterior surface of the thyroid gland. These glands are arranged in pairs, with two superior and two inferior glands. Each parathyroid gland measures approximately 6 mm in length, 3 mm in width, and 1 mm in thickness, weighing about 20-50 mg [6]. Their bean-shaped structure features a smooth, encapsulated surface that distinguishes them from the lobular structure of the thyroid gland. While the superior parathyroid glands are usually found near the upper pole of the thyroid, the inferior glands are near the lower pole. However, their exact positioning can vary among individuals [7].

The primary function of the parathyroid glands is to produce and secrete PTH, which is essential for maintaining calcium and phosphate balance in the bloodstream. When blood calcium levels drop, PTH secretion increases, triggering several physiological responses. PTH stimulates osteoclasts, the cells responsible for bone resorption, leading to the release of calcium from the bones into the bloodstream [8]. Additionally, PTH acts on the kidneys to promote calcium reabsorption while increasing phosphate excretion. It also stimulates the conversion of vitamin D into its active form, calcitriol, which enhances intestinal calcium absorption. Collectively, these actions ensure that blood calcium levels remain within a narrow range, crucial for various physiological processes, including muscle contraction, nerve function, and blood clotting [8].

The parathyroid glands have a close anatomical and functional relationship with the thyroid gland. This proximity is significant during surgical procedures, such as thyroidectomy, where there is a risk of inadvertently damaging or removing the parathyroid glands, potentially leading to complications such as hypoparathyroidism [9]. Embryologically, the parathyroid glands develop from the third and fourth pharyngeal pouches, contributing to their anatomical closeness and functional interdependence with the thyroid gland. This shared developmental pathway underscores the importance of careful surgical techniques to preserve parathyroid function during thyroid surgeries [9].

Pathophysiology of hypoparathyroidism

Postoperative hypoparathyroidism is a common complication following thyroidectomy, characterized by a deficiency in PTH, which is crucial for regulating calcium and phosphate levels in the body. The pathophysiology of hypoparathyroidism after thyroid surgery primarily involves surgical trauma to the parathyroid glands. This trauma can manifest in several ways, including accidental resection of parathyroid tissue, which is a significant risk during thyroid surgeries, especially in cases involving thyroid cancer or extensive neck dissection [10]. Additionally, the parathyroid glands are highly vascularized, making them susceptible to ischemic damage if their blood supply is compromised during surgery. Prolonged surgical duration, particularly when exceeding 120 minutes, has been correlated with an increased risk of hypoparathyroidism due to potential ischemia affecting the glands [10].

In some surgical cases, surgeons may attempt to preserve parathyroid function by autotransplanting parathyroid tissue. However, the success of this procedure can vary, and the transplanted glands may take time to regain functional capacity, contributing to transient hypoparathyroidism. The removal or damage to parathyroid glands results in decreased secretion of PTH, leading to hypocalcemia and hyperphosphatemia. This hormonal imbalance can cause a range of neuromuscular symptoms and other metabolic disturbances associated with hypoparathyroidism [11]. Transient hypoparathyroidism typically resolves within a few weeks to months following surgery. The incidence of transient hypoparathyroidism can be quite high, with reports indicating that up to 47.8% of patients may experience it within the first 24 hours post-surgery, decreasing to approximately 17.8% at three months [12]. Transient hypoparathyroidism often results from temporary ischemia or minor surgical trauma, where parathyroid function may recover as blood supply is restored and devascularized glands regain their functionality. Management of transient hypoparathyroidism usually involves calcium and vitamin D supplementation during the recovery period, with most patients eventually returning to normal PTH levels and calcium homeostasis [12].

In contrast, permanent hypoparathyroidism is defined as lasting beyond one year post-surgery, with reported incidences of approximately 10.7%. This form of hypoparathyroidism often results from the complete loss of parathyroid tissue due to resection or irreversible damage to the remaining glands, leading to chronic PTH deficiency. Patients with permanent hypoparathyroidism typically require lifelong calcium and vitamin D supplementation, and the condition can significantly impact their quality of life due to persistent symptoms of hypocalcemia [13]. Understanding the mechanisms and differences between transient and permanent hypoparathyroidism is essential for healthcare providers to anticipate, identify, and manage these conditions effectively following thyroid surgery. Continued research and improved surgical techniques aim to minimize the incidence of both forms of hypoparathyroidism, enhancing patient outcomes [14].

Incidence and prevalence

The incidence and prevalence of transient and permanent hypoparathyroidism following a total thyroidectomy exhibit significant variability based on patient demographics and surgical techniques. Transient hypoparathyroidism is relatively common after thyroid surgery, with reported incidence rates ranging from 10% to 46%. In some studies, transient hypoparathyroidism can be as high as 47.8% within the first 24 hours post-surgery, decreasing to approximately 17.8% at three months. A retrospective analysis highlighted that approximately 27.4% of patients experience transient hypoparathyroidism following a thyroidectomy. These statistics underscore the importance of monitoring calcium levels and parathyroid function in the immediate postoperative period [15].

In contrast, the prevalence of permanent hypoparathyroidism is generally lower, with estimates ranging from less than 1% to 10.6% across various studies. One study found that the incidence of permanent hypoparathyroidism was approximately 10.7% one year after surgery. Additionally, a cohort study indicated a prevalence of 10.3%, with a notable disparity between genders; females exhibited a higher prevalence (12.1%) than males (3.2%). This gender difference suggests that hormonal factors or anatomical variations may play a role in the susceptibility to hypoparathyroidism [16]. Demographic factors significantly influence the risk of hypoparathyroidism. Younger patients, particularly those under 40 years of age, are at a heightened risk of transient hypoparathyroidism. Furthermore, female patients have been shown to experience higher rates of both transient and permanent forms of the condition. These demographic variations highlight the need for tailored preoperative counseling and postoperative monitoring strategies [17].

Surgical techniques also play a crucial role in determining the incidence of hypoparathyroidism. The duration of surgery is a significant factor; longer procedures, especially those exceeding 120 minutes, are associated with increased risks of both transient and permanent hypoparathyroidism due to potential ischemic damage to the parathyroid glands [18]. Additionally, the practice of autotransplantation of parathyroid glands can lead to transient hypoparathyroidism but does not appear to significantly impact the rates of permanent hypoparathyroidism. Moreover, neck dissections, particularly when both central and lateral approaches are employed, further elevate the risk of hypoparathyroidism due to the potential for direct damage to the parathyroid glands during surgery [18].

Risk factors for transient hypoparathyroidism

Surgical Factors

Surgical factors play a crucial role in the incidence of transient hypoparathyroidism following thyroidectomy. One of the primary considerations is the extent of the thyroidectomy performed. Total thyroidectomy, which involves the complete removal of the thyroid gland, is associated with a higher risk of transient hypoparathyroidism compared to subtotal thyroidectomy [17]. The complete excision in total thyroidectomy increases the likelihood of damaging or inadvertently removing the parathyroid glands, essential for calcium regulation. Conversely, subtotal thyroidectomy, which preserves a portion of the thyroid gland, generally results in a lower incidence of transient hypoparathyroidism, as the remaining thyroid tissue can help maintain calcium homeostasis [17].

The surgeon's experience is another significant factor that influences outcomes. Surgeons who perform a higher number of thyroidectomies tend to achieve better results, including lower rates of complications such as transient hypoparathyroidism. This correlation can be attributed to the technical skill and familiarity that experienced surgeons develop over time. Their expertise enhances their ability to identify and preserve parathyroid glands during surgery, which is critical for minimizing the risk of postoperative hypoparathyroidism [19]. Intraoperative techniques for the identification and preservation of parathyroid glands are also vital. The ability to visually identify these glands during surgery is essential. Surgeons often employ careful dissection techniques and may use intraoperative nerve monitoring to aid in identifying parathyroid glands.

Furthermore, preserving the microvascular supply to these glands is crucial; damage to their blood supply can lead to transient hypoparathyroidism. A key component of successful thyroid surgery is ensuring that the parathyroid glands remain intact and well-perfused [20]. Lastly, intraoperative blood loss can significantly affect the risk of transient hypoparathyroidism. Significant blood loss during surgery can compromise the vascular supply to the parathyroid glands, increasing the likelihood of ischemia and subsequent gland dysfunction. Maintaining hemodynamic stability throughout the procedure ensures adequate blood flow to these critical glands. Surgeons must be vigilant in managing blood loss to mitigate this risk [16].

Patient Factors

Certain patient factors can increase the risk of transient and permanent hypoparathyroidism following thyroidectomy. Age and sex are two key demographic characteristics that influence the likelihood of developing hypoparathyroidism. Younger patients, particularly those under 40, are at a higher risk of transient hypoparathyroidism. This increased susceptibility may be due to the parathyroid glands being more prone to injury or dysfunction in younger individuals. Conversely, patients over 55 exhibit a lower risk, suggesting that age may play a protective role. Additionally, female patients have a higher prevalence of postoperative hypoparathyroidism compared to males [17]. Pre-existing parathyroid gland conditions can also predispose patients to a higher risk of hypoparathyroidism after thyroid surgery. Conditions such as parathyroid adenomas and hyperplasia can impair the function of the remaining parathyroid glands, making them more vulnerable to injury or removal during thyroidectomy. Patients who have undergone previous parathyroid surgery are also at an elevated risk of developing permanent hypoparathyroidism following thyroidectomy [17].

Autoimmune diseases can contribute to an increased risk of postoperative hypoparathyroidism. Hashimoto's thyroiditis, an autoimmune condition affecting the thyroid gland, has been associated with a higher risk of hypoparathyroidism, possibly due to the inflammatory process damaging the parathyroid glands. Other autoimmune conditions, such as type 1 diabetes, rheumatoid arthritis, and systemic lupus erythematosus, have also been linked to an increased risk of hypoparathyroidism after thyroidectomy [21]. Surgeons must know these patient-specific risk factors when planning and performing thyroid surgery. By identifying high-risk patients and taking appropriate precautions, the risk of postoperative hypoparathyroidism can be minimized, leading to improved patient outcomes and reduced complications following thyroidectomy [22].

Perioperative Factors

Perioperative factors play a crucial role in determining the risk of transient and permanent hypoparathyroidism following thyroidectomy. One of the most significant factors is the duration of surgery. Prolonged surgical time, particularly when exceeding 120 minutes, has been consistently linked to an increased risk of both transient and permanent hypoparathyroidism. Studies have shown that the incidence of hypoparathyroidism escalates with longer surgery durations, with rates reaching as high as 62% for procedures lasting over 240 minutes. This correlation suggests that longer surgical times may increase the likelihood of damage to the parathyroid glands or complicate their identification and preservation during the procedure [23].

Another important perioperative factor is the use of certain medications. Neuromuscular blocking agents (NMBAs) are commonly used during surgery but can pose risks if not managed properly. Improper use of NMBAs can interfere with intraoperative neuromonitoring (IONM) systems, which are essential for assessing the function of the parathyroid glands and recurrent laryngeal nerve during thyroid surgery. Ensuring the appropriate administration of these agents is vital to maintaining the integrity of IONM and minimizing the risk of postoperative complications [24].

**Figure 1:**
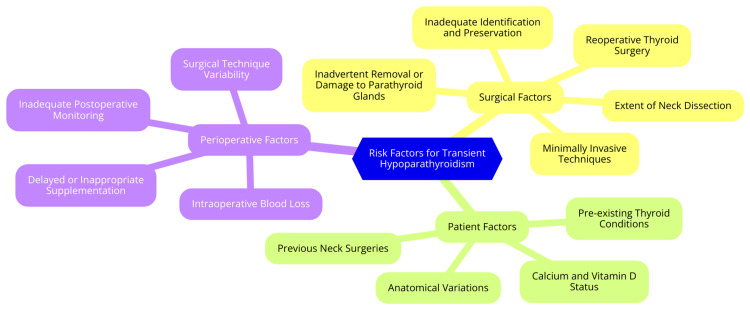
Risk factors for transient hypoparathyroidism Image credit: Dr. Maulik Maheshwari

Additionally, administering calcium and vitamin D supplements postoperatively is common in managing hypocalcemia. However, there is concern that failing to discontinue these supplements may lead to an overestimation of permanent hypoparathyroidism rates. Low calcium levels may persist due to ongoing supplementation rather than indicating true dysfunction of the parathyroid glands. Furthermore, the use of steroids during the perioperative period has been identified as a risk factor for complications in thyroid surgeries, potentially affecting parathyroid function [25]. The risk factors for transient hypoparathyroidism are shown in Figure 1.

Risk factors for permanent hypoparathyroidism

Surgical Factors

Surgical factors play a crucial role in the development of permanent hypoparathyroidism following a total thyroidectomy. One of the most significant risks is the unintentional removal or damage to the parathyroid glands during the procedure. When parathyroid glands are inadvertently excised, or their blood supply is compromised, the likelihood of permanent hypoparathyroidism increases substantially. Research indicates that the incidence of permanent hypoparathyroidism is markedly higher when one or more parathyroid glands are either removed or injured. Therefore, careful identification and preservation of these glands during surgery are essential to minimizing the risk of this complication [13]. Another important surgical factor is the history of repeated thyroid surgeries. Patients who undergo re-operative thyroid surgery are at a greater risk of developing permanent hypoparathyroidism compared to those undergoing their first thyroidectomy. The challenges associated with re-operative procedures include scarring and distortion of the anatomical structures, which can complicate the identification and preservation of the parathyroid glands. As the number of surgeries increases, so does the risk of PH, highlighting the need for careful surgical planning and technique in these cases [26]. The extent of neck dissection performed during thyroidectomy also significantly influences the risk of PH. Central neck dissection (CND), in particular, is associated with a higher incidence of permanent hypoparathyroidism due to the increased likelihood of parathyroid gland removal or vascular compromise [27]. Additionally, lateral neck dissection, especially when conducted bilaterally, further elevates the risk of injury to the parathyroid glands. As the extent of neck dissection increases, so does the potential for damage to these critical glands, underscoring the importance of weighing the benefits of extensive dissection against the risks of hypoparathyroidism [27].

Patient Factors

Genetic predispositions can significantly influence the risk of hypoparathyroidism. Certain patients may inherit conditions that predispose them to parathyroid gland dysfunction, such as familial hypoparathyroidism, often associated with syndromes such as DiGeorge syndrome or autoimmune polyglandular syndrome. These genetic conditions can lead to a higher likelihood of developing permanent hypoparathyroidism after thyroid surgery [28]. Additionally, specific genetic mutations, particularly those affecting the calcium-sensing receptor (CaSR), can increase susceptibility to hypoparathyroidism. Therefore, understanding a patient's family history and potential genetic markers is essential for identifying individuals at greater risk [28]. Comorbid conditions also contribute to the risk of PH. Chronic kidney disease (CKD) is a notable example, as it can alter calcium and phosphate metabolism, complicating the postoperative management of calcium levels. Patients with CKD often experience secondary hyperparathyroidism, and the surgical removal of the thyroid can exacerbate the risk of developing permanent hypoparathyroidism [29]. Furthermore, autoimmune disorders, including autoimmune polyglandular syndrome, can affect parathyroid function, increasing susceptibility to hypoparathyroidism post-thyroidectomy. Additionally, a history of previous neck surgeries may compromise parathyroid gland function, further elevating the risk during subsequent thyroid operations [29]. Prior radiation therapy is another critical factor influencing the development of PH. Patients who have received radiation treatment to the neck for conditions such as head and neck cancers face a significantly increased risk of permanent hypoparathyroidism [30]. Radiation can cause fibrosis and damage to the parathyroid glands, making them more vulnerable to injury during thyroid surgery. The effects of radiation often lead to vascular damage, reducing blood supply to the parathyroid glands and increasing the likelihood of ischemic injury during surgical manipulation [30].

Perioperative Factors

The most effective perioperative strategies for managing postoperative hypocalcemia and enabling early intervention and monitoring include closely monitoring postoperative PTH levels, administering perioperative calcium and vitamin D supplements, and tracking perioperative calcium level changes [31]. Patients undergoing total thyroidectomy or parathyroidectomy who have eight-hour postoperative PTH levels less than 15 pg/mL (1.6 pmol/L) are at a very high risk of developing postoperative hypocalcemia [32]. These patients should monitor their serum calcium levels closely and be considered for early oral calcium and vitamin D supplementation administration.

**Figure 2:**
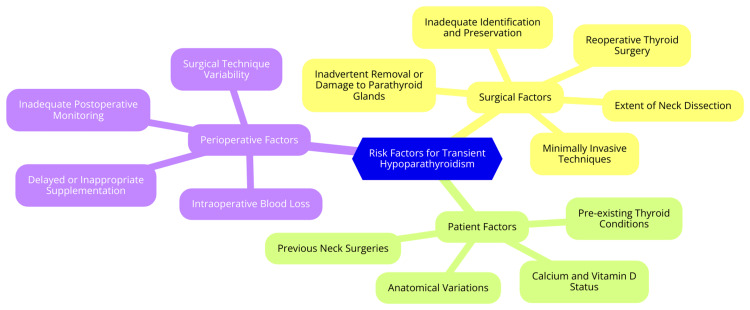
Risk factors for permanent hypoparathyroidism Image credit: Dr. Maulik Maheshwari

Compared to postoperative supplementation alone, perioperative oral calcium and vitamin D supplementation significantly decreases the risks of symptomatic and biochemical hypocalcemia. Perioperative supplementation also shortens the recovery period of symptomatic hypocalcemia to within 24 hours [33]. A drop in serum calcium levels on postoperative day one of more than 1.1 mg/dL compared to preoperative levels is a strong predictor of hypocalcemia. Evaluating the trend of calcium changes postoperatively can aid in predicting subsequent hypocalcemia and identifying patients at risk who may need early calcium and vitamin D replacement [34]. Close monitoring of postoperative PTH levels, administering perioperative calcium and vitamin D supplements, and tracking perioperative calcium level changes are key factors for the early identification of patients at risk of hypocalcemia. This enables timely intervention with oral supplements or intravenous calcium infusion to manage hypocalcemia and its symptoms [34]. The risk factors for permanent hypoparathyroidism are shown in Figure 2.

Diagnostic and monitoring strategies

Preoperative assessment is crucial for optimizing patient outcomes following thyroidectomy. This process starts with a thorough evaluation of the patient's medical history and a physical examination of the thyroid gland to identify any risk factors that may complicate surgery or recovery. Laboratory tests should be conducted, including baseline serum calcium and thyroid function tests (thyroid-stimulating hormone (TSH), free T4, and T3) [35].

Additionally, assessing vitamin D levels is important to address potential deficiencies preoperatively, as low vitamin D can contribute to postoperative hypocalcemia. Imaging techniques, such as thyroid ultrasound and fine-needle aspiration biopsy, are also employed to evaluate nodules and assess the anatomical relationship of the thyroid to surrounding structures, including the recurrent laryngeal nerve [35]. Intraoperative monitoring is essential for preventing complications, particularly hypoparathyroidism and recurrent laryngeal nerve injury. One key technique is continuous IONM of the recurrent laryngeal nerve, which uses an electromyography device to monitor nerve function during surgery [36]. This real-time feedback helps surgeons avoid damaging the nerve. Additionally, surgeons are trained to visually identify and preserve the parathyroid glands during thyroidectomy. This meticulous approach is crucial for preventing postoperative hypoparathyroidism, as the inadvertent removal or devascularization of these glands can lead to significant complications [36].

Postoperative monitoring of calcium and PTH levels is vital for the early detection of hypoparathyroidism. Patients should have their serum calcium levels checked within the first 24 hours after surgery and subsequently at regular intervals, especially if they exhibit symptoms of hypocalcemia, such as tingling or muscle cramps [37]. Monitoring PTH levels can also provide valuable insights; a low PTH level alongside low calcium indicates hypoparathyroidism, while normal PTH levels with low calcium may suggest other underlying issues. This proactive approach allows timely intervention and management of potential complications [37]. Various imaging modalities can assist in identifying parathyroid glands preoperatively, enhancing surgical planning and outcomes. Neck ultrasound is the most commonly used imaging technique to locate parathyroid glands and assess their vascularity and relationship to thyroid nodules [38]. Additionally, sestamibi scintigraphy, a nuclear medicine technique, can help localize hyperfunctioning parathyroid glands, particularly in cases of primary hyperparathyroidism, although its role in thyroidectomy planning is less common. In more complex cases, such as when there is suspicion of ectopic parathyroid tissue or significant anatomical variations, computed tomography (CT) and magnetic resonance imaging (MRI) may be indicated [38].

Management of transient hypoparathyroidism

The management of transient hypoparathyroidism following thyroidectomy primarily involves calcium and vitamin D supplementation. After surgery, patients typically require oral calcium and vitamin D to address postoperative hypocalcemia [25]. Commonly used supplements include calcium carbonate or calcium gluconate, administered in doses of 1 to 3 g of elemental calcium per day, divided into multiple doses. Additionally, active vitamin D, such as calcitriol, is usually prescribed at doses ranging from 0.25 to 1 µg twice daily. Dosages are carefully adjusted based on the patient's serum calcium levels and clinical symptoms [25]. Monitoring and adjusting therapy are crucial for managing transient hypoparathyroidism. Serum calcium levels are monitored daily after surgery until they stabilize, with the supplementation regimen modified according to the patient's symptoms and laboratory results [4]. No treatment is necessary if a patient is asymptomatic with normal calcium levels. However, if the patient is asymptomatic but has low calcium levels, the calcium and vitamin D doses may increase. In cases where patients exhibit symptoms of hypocalcemia, such as muscle cramps or paresthesias, intravenous calcium gluconate may be administered for rapid relief. As the patient's condition improves, they gradually transition to oral therapy [4].

Patient education and follow-up are essential components of managing transient hypoparathyroidism. Patients should be informed about the signs and symptoms of hypocalcemia, which can include tingling sensations, muscle spasms, and, in severe cases, seizures. They need to understand how to take their supplements effectively, such as consuming them with meals and avoiding calcium-binding antacids that could interfere with absorption [4]. Regular follow-up appointments are necessary, with serum calcium levels initially checked one to two times per week. Most patients can expect to discontinue supplements within one to six months, as the restoration of parathyroid function is frequently observed. If hypocalcemia persists beyond one year, it may indicate permanent hypoparathyroidism, necessitating further evaluation and management [4].

Management of permanent hypoparathyroidism

Permanent hypoparathyroidism requires a comprehensive management strategy to maintain calcium homeostasis and prevent complications. The cornerstone of treatment involves lifelong calcium and vitamin D supplementation [39]. Patients typically need a daily calcium intake of around 1,000 to 1,500 mg of elemental calcium to maintain normal serum calcium levels. Alongside calcium, active forms of vitamin D, such as calcitriol (1,25-dihydroxyvitamin D), are crucial for enhancing calcium absorption from the gastrointestinal tract. Vitamin D dosage is individualized based on serum calcium and PTH levels, usually starting at 0.25 to 1.0 µg daily [39]. If calcium and vitamin D supplementation alone are insufficient to maintain normal serum calcium levels, PTH replacement therapy may be considered. Recombinant PTH (teriparatide) can be administered subcutaneously to better regulate calcium levels. Patients on PTH therapy require regular monitoring of serum calcium and renal function to avoid complications such as hypercalcemia and kidney issues [40].

Lifestyle modifications and patient education are also crucial. Patients should be informed about dietary sources of calcium, such as dairy products, leafy greens, and fortified foods, and the importance of maintaining adequate intake. Adequate hydration is essential to prevent kidney stones resulting from elevated calcium levels [41]. Regular, moderate exercise is encouraged to improve bone health and overall well-being, although high-impact activities should be approached with caution due to fracture risk. Educating patients about their condition, treatment adherence, and recognizing hypocalcemia symptoms, such as tingling and muscle cramps, is vital for effective self-management [42]. Regular follow-up appointments are necessary to monitor serum calcium, phosphate, and vitamin D levels and renal function. Periodic bone density scans (DEXA) may be warranted to assess bone health, as patients with permanent hypoparathyroidism are at increased risk of osteoporosis. Additionally, patients should be informed about potential complications, such as kidney stones and cardiovascular issues related to calcium imbalance, so they can seek timely medical attention [43].

Prevention strategies

Preventing transient and permanent hypoparathyroidism following thyroidectomy requires a multifaceted approach that includes advanced surgical techniques, the expertise of the surgeon, intraoperative monitoring, and meticulous patient selection. Each element is crucial in minimizing the risk of complications associated with thyroid surgery [26]. One of the primary strategies for prevention involves the surgical techniques used during the procedure. Surgeons should prioritize the identification and preservation of all four parathyroid glands. Meticulous dissection and magnification techniques enhance visibility and reduce the likelihood of damaging these critical structures [11]. In cases where a parathyroid gland is inadvertently removed or damaged, autotransplantation into a different site, such as the sternocleidomastoid muscle, can help preserve parathyroid function. Additionally, minimally invasive surgical techniques, such as endoscopic or robotic-assisted thyroidectomy, can reduce trauma to surrounding tissues and further protect the parathyroid glands [11]. The expertise and training of the surgeon play a significant role in preventing hypoparathyroidism. Surgical outcomes are notably better when procedures are performed by experienced surgeons who regularly conduct thyroid surgeries [44]. The volume of surgeries correlates with the surgeon's skill in preserving parathyroid function. Ongoing education and specialized training in advanced surgical techniques, including a thorough understanding of parathyroid anatomy, are essential for minimizing complications and enhancing surgical proficiency [44].

IONM is a valuable tool during thyroidectomy. By utilizing IONM, surgeons can identify the recurrent laryngeal nerve and assess the functional status of the parathyroid glands in real time. This feedback enables informed decision-making and adjustments during surgery, significantly reducing the risk of damage to these critical structures. Some centers also explore intraoperative PTH monitoring to evaluate gland viability and further guide surgical interventions [45]. Careful patient selection and preoperative planning are also vital in preventing hypoparathyroidism. A comprehensive assessment of the patient's medical history, thyroid pathology, and previous neck surgeries is essential [46]. Patients with conditions such as Graves' disease or those who have undergone prior thyroid surgeries may require tailored surgical approaches to minimize risk. Educating patients about the potential risks of hypoparathyroidism and the importance of selecting a skilled surgeon can help set realistic expectations and encourage adherence to postoperative follow-up. A multidisciplinary approach involving collaboration among endocrinologists, surgeons, and anesthesiologists can enhance preoperative planning and optimize patient outcomes [46].

Emerging research and future directions

Recent advancements in surgical techniques have significantly improved the preservation of parathyroid glands during thyroid surgeries. Techniques such as near-infrared autofluorescence (NIR-AF) and indocyanine green (ICG) angiography have shown great promise in the real-time identification of parathyroid glands, enhancing the ability to distinguish them from surrounding tissues and reducing the risk of damage during surgery [47]. These methods facilitate visualization of the blood supply to the glands, which is crucial for their viability post-surgery. Additionally, the use of carbon nanoparticles and optical technologies has been explored to further enhance intraoperative identification of parathyroid glands, potentially leading to better outcomes in preserving parathyroid function [47]. Intraoperative imaging techniques have advanced notably, with fluorescence imaging emerging as a key tool for identifying and preserving parathyroid glands. This approach has been validated in vivo and in vitro, enabling surgeons to visualize parathyroid glands more effectively during procedures. The integration of these imaging technologies aims to decrease the incidence of postoperative complications such as hypoparathyroidism, which can occur in 20%-35% of patients undergoing thyroidectomy [48].

Ongoing research is delving into the genetic and molecular factors contributing to individual susceptibility to hypoparathyroidism following thyroid surgery. Understanding these factors may lead to developing targeted interventions to mitigate risks. Genetic studies could identify specific markers predicting postoperative outcomes, allowing for personalized surgical approaches based on a patient's genetic profile [17]. Future therapeutic advancements may include the development of pharmacological agents to enhance parathyroid function or protect against ischemic damage during surgery. Additionally, parathyroid autotransplantation remains a viable option for cases where glands are inadvertently removed, and research is exploring various preservation solutions to maintain the viability of resected glands before transplantation [49].

## Conclusions

In conclusion, hypoparathyroidism remains a significant and challenging complication following thyroidectomy, with profound implications for patients' long-term health and quality of life. This comprehensive review highlights that a complex interplay of surgical, patient-related, and perioperative factors influences both transient and permanent forms of hypoparathyroidism. The extent of thyroidectomy, the surgeon's experience, and intraoperative techniques significantly impact the risk of hypoparathyroidism. Patient factors such as age, pre-existing conditions, and genetic predispositions also play a crucial role. Effective strategies for prevention and management include meticulous surgical techniques, improved intraoperative monitoring, and personalized postoperative care protocols. By advancing our understanding of these risk factors and integrating evidence-based practices, healthcare professionals can better mitigate the incidence of hypoparathyroidism, thereby enhancing patient outcomes. Future research should continue to explore innovative surgical methods, advanced monitoring technologies, and novel therapeutic approaches to further reduce the burden of this complication.
